# Short Term Effect of Salt Shock on Ethylene and Polyamines Depends on Plant Salt Sensitivity

**DOI:** 10.3389/fpls.2017.00855

**Published:** 2017-05-23

**Authors:** Pedro J. Zapata, María Serrano, Manuel F. García-Legaz, M. T. Pretel, M. A. Botella

**Affiliations:** ^1^Departamento de Tecnología Agroalimentaria, Universidad Miguel HernándezOrihuela, Spain; ^2^Departamento de Biología Aplicada, Universidad Miguel HernándezOrihuela, Spain; ^3^Departamento de Agroquímica y Medioambiente, Universidad Miguel HernándezOrihuela, Spain

**Keywords:** ethylene, ACC, respiration, polyamines, salinity, salinity tolerance

## Abstract

In the present manuscript the short term effect (3–24 h) of a saline shock (NaCl 100 mM) on fresh weight, water content, respiration rate, ethylene production and Na^+^, Cl^-^, ACC and polyamine concentration was studied in four plant species with different salt sensitivity, pepper, lettuce, spinach, and beetroot. Higher reduction in fresh weight and water content as a consequence of saline shock was found in pepper and lettuce plants than in spinach and beetroot, the latter behaving as more salinity tolerant. In general, salinity led to rapid increases in respiration rate, ethylene production and ACC and polyamine (putrescine, spermidine, and spermine) concentrations in shoot and root. These increases were related to plant salinity sensitivity, since they were higher in the most sensitive species and *vice versa*. However, ethylene and respiration rates in salt stressed plants recovered similar values to controls after 24 h of treatment in salt tolerant plants, while still remaining high in the most sensitive. On the other hand, sudden increases in putrescine, spermidine, and spermine concentration were higher and occurred earlier in pepper and lettuce, the most sensitive species, than in spinach and beetroot, the less sensitive ones. These increases tended to disappear after 24 h, except in lettuce. These changes would support the conclusion that ethylene and polyamine increases could be considered as a plant response to saline shock and related to the plant species sensitivity to this stress. In addition, no competition between polyamines and ethylene biosynthesis for their common precursor was observed.

## Introduction

Salinity in soils is harmful to most plants and limits crop production. Understanding the mechanisms of plant salt tolerance and adaptation is important for developing new approaches to enhance stress tolerance (Liu et al., 2015). Salt stress affects plants, leading to changes at different levels, morphological, physiological, biochemical, and molecular, and including increases in ethylene biosynthesis and in the concentration of its precursor 1-aminocyclopropane-1-carboxylic acid (ACC). Thus, in some halophyte species under high salinity, ethylene production, and ACC concentration increased in leaves and roots (Ellouzi et al., 2014). In tolerant soybean genotypes these increments were higher than in salt sensitive ones (Ma et al., 2012). In addition, the application of ethylene or ACC enhanced Arabidopsis plant tolerance to salt stress throughout increases in the expression of genes involved in scavenging reactive oxygen species (ROS) (Peng et al., 2014), while inhibition of ethylene biosynthesis leads to increased plant sensitivity to salinity (Tao et al., 2015). These studies, as well as others related to gene mutation and transformation analysis, indicated that plant tolerance to salinity could be enhanced by the ethylene biosynthesis and signal transduction pathway (Tao et al., 2015). Accordingly, previous experiments by our group found an increase in ethylene biosynthesis induced by salinity in several cultivars of lettuce during the germination phase, although cultivars showing the most salt sensitivity (highest decrease in fresh weight) showed the lowest increase in ethylene production and *vice versa* (Zapata et al., 2003). These results suggest that the ability to increase the ethylene production under saline conditions could provide a higher tolerance to salinity during lettuce germination.

However, when comparing germination percentage and seedling growth of different plant species (lettuce, pepper, broccoli, beetroot, melon, spinach, and tomato) under saline conditions a general effect of salinity on ethylene metabolism during germination could not been found (Zapata et al., 2004). In a different approach, when saline treatment (NaCl 100 mM) was applied to plants progressively (to avoid osmotic shock) and with long term exposition to salt stress total ACC concentration increased in pepper tomato, broccoli, lettuce, melon, bean, spinach and beetroot, this increase being higher in the species most sensitive to salinity. The plant species most sensitive to saline treatment was pepper, with the highest reduction in fresh weight and the highest increase in total ACC concentration occurring. On the other hand, the least affected by salinity was beetroot which did not present changes in total ACC concentration following salt treatment (Zapata et al., 2007). These discrepancies on ethylene responses to saline treatment during germination and more developed plants could be attributed to changes on salt sensitivity during plant development. Interestingly, broccoli was found to be more saline tolerant than pepper, melon and lettuce, but it was more affected by salinity than the other plant species during the germination phase (Zapata et al., 2004).

The biosynthesis pathway of polyamines is related with that of ethylene biosynthesis. *S*-adenosylmethionine (SAM) can be used to form ACC, the precursor of ethylene and in the conversion of putrescine (Put) into spermidine (Spd) and of Spd into spermine (Spm) by two reactions catalyzed by Spd-synthase and Spm synthase respectively. Different abiotic stresses, such as low and high temperatures, drought, high salinity, and nutrient deficiency have been widely shown to produce changes in polyamine levels in a number of plant species (Liu et al., 2007; Kusano et al., 2008; Alcázar et al., 2010; Minocha et al., 2014; Tiburcio et al., 2014). In this sense, it has been proposed that polyamines have a role in plant adaptive responses to various environmental stresses since the expression of the genes involved in their biosynthesis as well as their concentration increase under stress abiotic conditions and exogenous application of Put, Spd, or Spm enhances tolerance to these stresses (Pillai and Akiyama, 2004; Duan et al., 2008; Wang B. Q. et al., 2011; Wang J. et al., 2011; Liu et al., 2015). The increase in polyamine levels under saline conditions can be considered an important adaptive mechanism as polyamines may modulate the activity of plasma membrane ion channels, reducing the uptake of Na^+^ and the leakage of K^+^ from mesophyll cells, therefore polyamines assist plants in their adaptation to salinity (Shabala et al., 2007). In addition, in forest trees polyamines have been considered as a possible biochemical marker for persistent environmental stress when phenotypic symptoms of stress are not yet visible (Jouve et al., 2004; Minocha et al., 2014). However, in rice salt sensitive cultivars showed higher contents of Put under control conditions, while under long term salinity Put levels decreased. These changes were correlated to salt sensitivity (Do et al., 2014).

Although polyamine accumulation is considered as a general plant response to abiotic stresses, the cause-effect relationship between PA accumulation and protection still remains unclear (Minocha et al., 2014). In fact, different results on polyamine accumulation have been obtained depending on plant species, physiological status of the examined tissues/organs, experimental conditions, short or long exposition to stress, or if the stress arises suddenly or slowly (Zapata et al., 2008; Minocha et al., 2014; Liu et al., 2015). For instance, the effect of polyamines on both K^+^ and H^+^ transport activities in the plasma membrane in maize and Arabidopsis roots was found to be specific to species, tissues, and growth conditions (Pandolfi et al., 2010).

The objective of this research was to study, by using the whole plant, the short term effect (hours) of a saline shock on ethylene biosynthesis and polyamine accumulation in four different plant species with different salt sensitivity, in order to find if immediate changes in ethylene or polyamines are related to plant salinity tolerance.

## Materials and Methods

Experiments were made with four plant species: pepper (*Capsicum annuum* L. cv. Pairal), lettuce (*Lactuca sativa* var. Longifolia Lam. cv. Inverna), spinach (*Spinacia oleracea* L. cv. Boeing) and beetroot [*Beta vulgaris* L. var. Crassa (Alef.) J. Helm. cv. Detroit]. The different seeds were provided by the following companies: spinach by Seminis Vegetable Seeds Iberica S.A. (Cartagena, Spain); lettuce by Battle S.A. (Madrid, Spain); pepper by Semillas Fitó (Barcelona, Spain); beetroot by Intersemillas (Valencia, Spain).

Seeds were sterilized by dipping in 5% sodium hypochlorite for 5 min. Afterward they were washed thoroughly with distilled water and germinated in vermiculite moistened with 0.5 mM Ca_2_SO_4_ in a germination chamber, adding distilled water when necessary. Seeds were under dark conditions in the germination chamber and at the optimum temperature for each plant species, that is 20°C for spinach, lettuce, and beetroot and 25°C for pepper. The seedlings were transferred to a growth chamber when cotyledons had fully emerged, after 3 days in lettuce and 4 days in spinach, pepper, and beetroot and were maintained in optimum conditions depending on plant species: 20/16°C for spinach, lettuce, and beetroot, and 28/22°C for pepper, a 16/8 h light/dark cycle, a relative humidity of 60% (day) and 80% (night) and with a photon flux density of 450 μmol m^-2^ s^-1^. After 10 days the seedlings were transplanted to 13-L pots with half strength Hoagland nutrient solution. The pH was adjusted daily to 5.5–6.0 and the solutions were renewed every 3 days. Two treatments were applied, a control (NaCl 1 mM) and a saline (NaCl 100 mM), the later suddenly applied when plants were 11 days with nutrient solution, in order to induce a saline shock.

After 0, 3, 6, 12, and 24 h of saline shock application nine random plants per replicate per treatment were taken, but at 12 h of the control no plants were taken. Plants of each of the four replicates were separated into root and shoot and were used to determine fresh weight, respiration rate and ethylene production. Later, the nine plants of each replicate were divided into groups of three plants, one of them being used for water content and Na^+^ and Cl^-^ quantifications and the remainder frozen and ground in liquid N_2_, with one of these used to quantify free and total ACC concentration and the other for polyamines quantification.

### Analytical Determinations

Fresh weight, dry weight, and water content were recorded in shoot and root of each individual plant from the four replicates. Results were expressed as g per plant and were the mean ± SE of four replicates for each plant species and treatment.

Ethylene production and respiration rates were determined by placing the shoots or roots of each replicate, corresponding to nine plants, in a glass jar of appropriated volume hermetically sealed with a rubber stopper. One mL of holder atmosphere was extracted after 1 h which was injected into Hewlett Packard 5890 Series II gas chromatograph, equipped with a flame ionization detector, and a 3-m stainless steel column with an inner diameter of 3.5 mm containing activated alumina of 80/100 mesh to quantify ethylene production. Column temperature was 90°C and injector and detector temperature 150°C. Ethylene was expressed in nanoliters evolved per gram of tissue per hour (nL g^-1^ h^-1^) and results are the mean ± SE of triplicate measurements in each of the four replicates for each plant species and treatment. Another mL of the same atmosphere was used to determine respiration rate, monitoring the CO_2_ concentration in a Shimadzu 14-A gas chromatograph with catarometric detector. Column temperature was 50°C. Respiration rate was expressed as μg of CO_2_ evolved per gram of tissue per hour (μg g^-1^ h^-1^) and results are the mean ± SE of triplicate measurements in each of the four replicates for each plant species and treatment.

Total ACC (free and conjugated) was extracted as previously described (Zapata et al., 2004). Shoot or root tissue from each sample was ground in a mortar with a pestle by using 0.2 M trichloroacetic acid (1:3 w/v) and the homogenate was centrifuged at 7000 *g* for 10 min. The supernatant was used to determine its free-ACC content by chemical conversion of ACC to ethylene in a reaction medium containing 100 μL of 10 mM ClHg_2_, 100 μL of saturated NaOH, 0.5 mL of 5% NaOCl and 0.5 mL of extract. Five mL of the same extract were mixed with 1 mL of 2 N HCl, at 100°C for 1 h in order to hydrolyse conjugated ACC. Then total AAC was measured as described above and conjugated ACC quantified as total ACC minus free ACC. In both cases, measurements were made in triplicate and a calibration curve of ACC from Sigma (Poole, Dorset, England) was used as standard. Results were expressed as nmol per gram of fresh weight (nmol g^-1^ FW) and are the mean ± SE of triplicate measurements in each of the four replicates for each plant species and treatment.

Plant material was dried at 65°C for 4 days and dry weight determined. Dried matter from root and shoot was ground and sieved to 0.5 mm diameter and dried material was digested with HNO_3_:HClO_4_ (2:1, v:v). Na^+^ was quantified by atomic absorption spectrometry by using a Perking Elmer spectrophotometer (mod. ICP-5000). Cl^-^ was extracted from dry material with HNO_3_ 0.1 N and glacial acetic acid (10%) and keeping in mechanical agitation for 2 h. Samples were quantified in duplicate by titration using an automatic titrater 702 SM Titrino model.

Free polyamines were extracted by homogenizing 1 g of shoot or root samples in 10 ml of 5% perchloric acid using a mortar and pestle. After that the homogenate was centrifuged for 30 min at 20000 *g*. Free polyamines in the supernatant were quantified after benzoilation by using a HPLC (Hewlett-Packard Company, Wilmington, DE, USA) system equipped with a reversed-phase column (LiChroCart 250–4.5 μm) and absorbance detector at 254 nm. The elution system was MeOH/H_2_O (64:36) running isocratically with a flow rate of 0.8 mL min^-1^ (Zapata et al., 2003). A relative calibration procedure using 1,6-hexanediamine (100 nmol g^-1^ FW of tissue) as an internal standard, and standard curves of Put, Spd, and Spm from Sigma (Poole, Dorset, England) was used to determine the polyamines in samples. The results are expressed as nmols per gram of fresh weight (nmol g^-1^ FW) and are the mean ± SE of quantifications performed in duplicate in each of the four replicates per treatment.

### Statistics

All data were statistically analyzed by ANOVA and a Student’s *t*-test was performed for shoots and roots in each species to find out significant differences between control and saline treatments at *p* < 0.05.

## Results

### Plant Growth and Water Content

**FIGURE 1:**
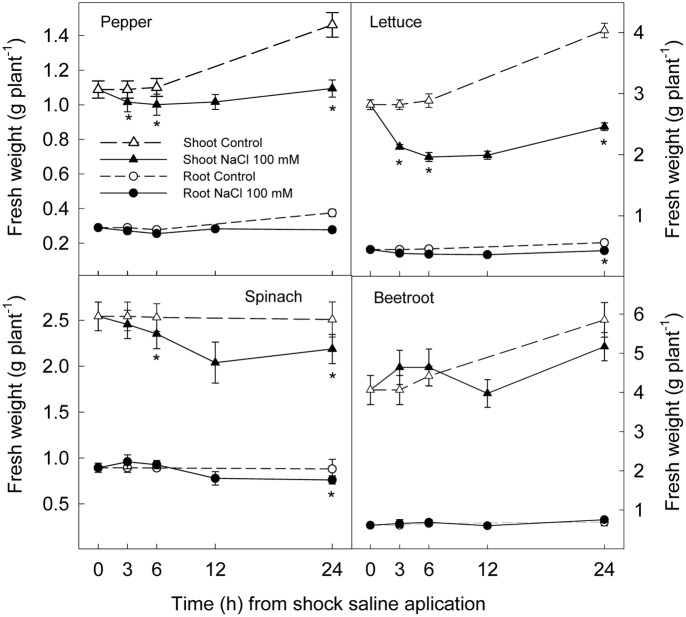
**Effect of saline shock (100 mM NaCl) on shoot and root fresh weight of different plant species**. Data are the mean ± SE of four replicates of nine plants. ^∗^Shows significant differences between control and saline treatment for each sampling date.

**FIGURE 2:**
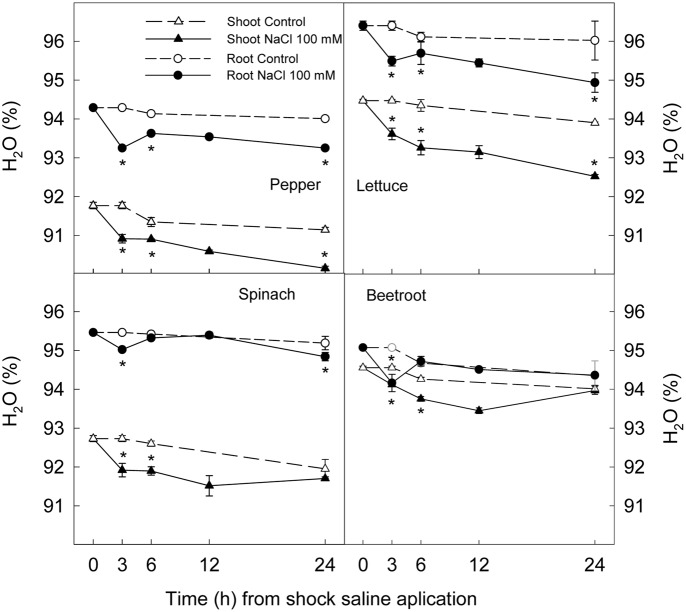
**Effect of saline shock (100 mM NaCl) on shoot and root water content of different plant species**. Data are the mean ± SE of four replicates of three plants. ^∗^Shows significant differences between control and saline treatment for each sampling date.

The application of a saline shock (NaCl 100 mM) decreased shoot and root fresh weight, although with significant differences among plant species. Thus, 24 h after NaCl application shoot fresh weight was reduced by 39 and 25% in lettuce and pepper respectively, while no significant reduction was found in spinach or beetroot (**Figure 1**). Root fresh weight after 24 h was reduced ≈25% in lettuce and pepper and ≈15% in spinach, while in beetroot no significant effect of saline application in root fresh weight was found (**Figure 1**). Water content was reduced by 1% in shoots and roots after 3 h of stress in all studied plant species, indicating a lower water uptake. However, after 6 h, a recovery was observed in root of beetroot and spinach, and at 24 h in the shoot of these plants. Contrarily, in pepper and lettuce plants water content was still lower in treated plants than in the control at the end of the experiments (**Figure 2**). These results show that pepper and lettuce behaved as more sensitive to saline shock, and spinach and beetroot as more tolerant.

### Concentration of Na^+^ and Cl^-^

**FIGURE 3:**
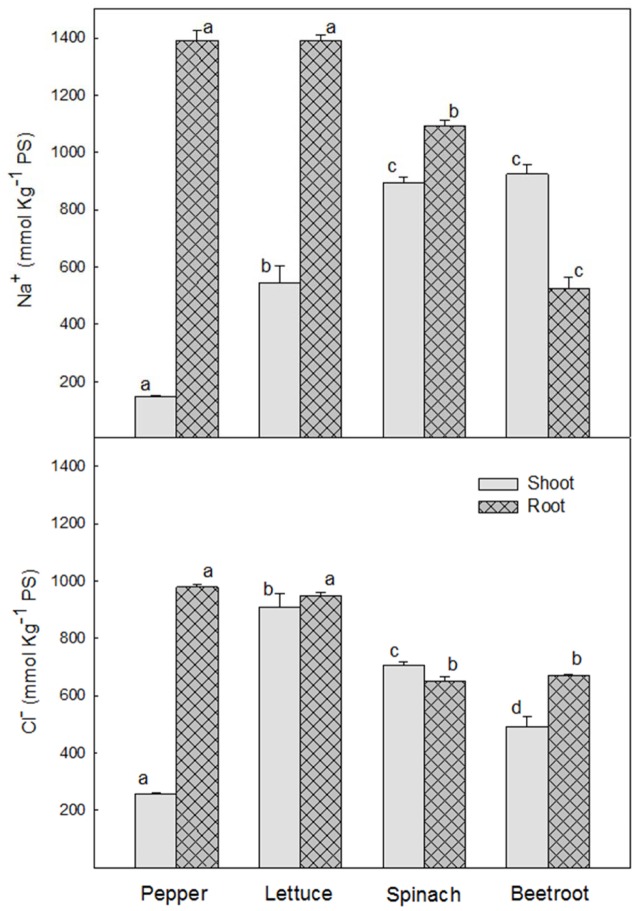
**Sodium and chloride concentration, in shoot and root of the different plant species, after 24 h of saline shock application**. Data are the mean ± SE of determinations made independently in four replicates of three plants. Different letters represent significant differences among plant species at *p* < 0.05.

Levels of Na^+^ and Cl^-^ were very low under control conditions. Thus, only data from saline treatments are presented. After 24 h of saline treatment Na^+^ concentration was significantly higher in the roots of the most sensitive species, pepper and lettuce (1392 ± 35 and 1391 ± 19 mmol Kg^-1^ DW, respectively) than in the most tolerant ones, spinach and beetroot (1092 ± 19 and 526 ± 40 mmol Kg^-1^ DW, respectively), while in shoots Na^+^ concentration was higher in the latter (894 ± 20 and 924 ± 35 mmol Kg^-1^ DW in spinach and beetroot, respectively), and very low in pepper (**Figure 3**).

Cl^-^ concentration in roots was also higher in the most sensitive species (≈950 mmol Kg^-1^ DW) than in the most tolerant (≈560 mmol Kg^-1^ DW). However, in shoots the two most sensitive species, pepper and lettuce, behaved in a different way, the highest Cl^-^ concentration being found in lettuce (≈1000 mmol Kg^-1^ DW), followed by spinach (≈700 mmol Kg^-1^ DW) and beetroot (≈500 mmol Kg^-1^ DW). Pepper showed the lowest (≈250 mmol Kg^-1^ DW). As can be seen, Cl^-^ concentration was similar in root and shoot from lettuce, spinach and beetroot, while in pepper it accumulated mainly in the root, with very little in the shoot (**Figure 3**).

### Respiration Rate

**FIGURE 4:**
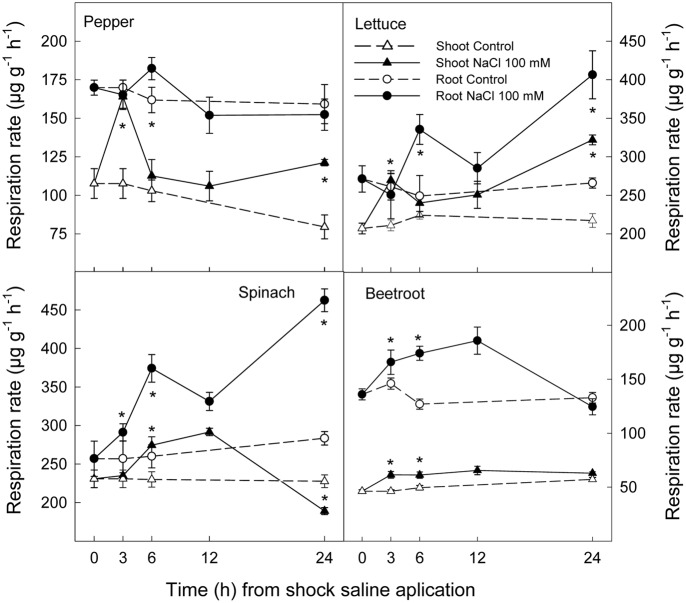
**Effect of saline shock (100 mM NaCl) on shoot and root respiration rate of different plant species**. Data are the mean ± SE of four replicates of nine plants. ^∗^Shows significant differences between control and saline treatment for each sampling date.

Saline shock led to a significant increase in respiration rate in both shoots and roots of all plant species, although the time and intensity of this increase was dependent on species. Thus, in shoot of pepper and lettuce this increase was evident after 3 h of saline shock, decreased to values similar to controls after 6 and 12 h and increasing again thereafter. However, in spinach and beetroot shoot the increase in respiration rate as a consequence of saline shock occurred later, after 6 h, and reached similar values to control plants after 24 h. With respect to root tissues, respiration rate also increased in all plants species, although after 24 h of saline shock application these effects disappeared in pepper and beetroot. However, in lettuce and spinach the highest differences between control and treated plants were found at this time (**Figure 4**).

### Ethylene Production and ACC Concentration

**FIGURE 5:**
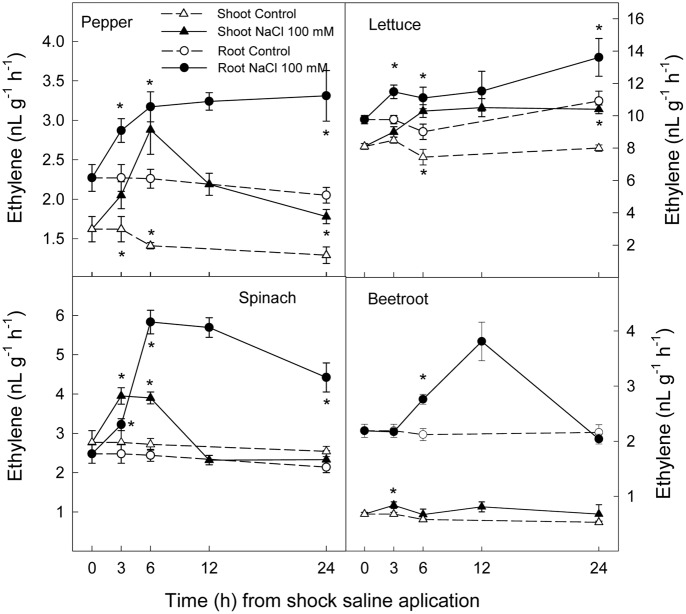
**Effect of saline shock (100 mM NaCl) on shoot and root ethylene production of different plant species**. Data are the mean ± SE of four replicates of nine plants. ^∗^Shows significant differences between control and saline treatment for each sampling date.

**FIGURE 6:**
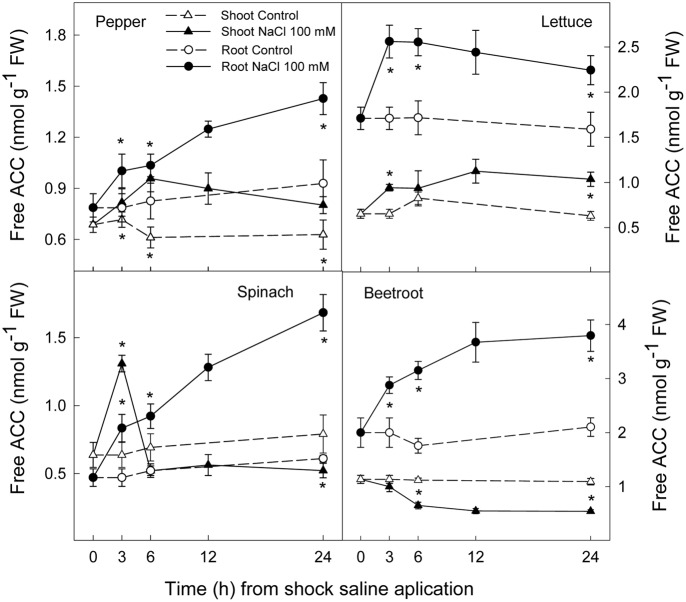
**Effect of saline shock (100 mM NaCl) on shoot and root free ACC concentration in different plant species**. Data are the mean ± SE of four replicates of three plants. ^∗^Shows significant differences between control and saline treatment for each sampling date.

**FIGURE 7:**
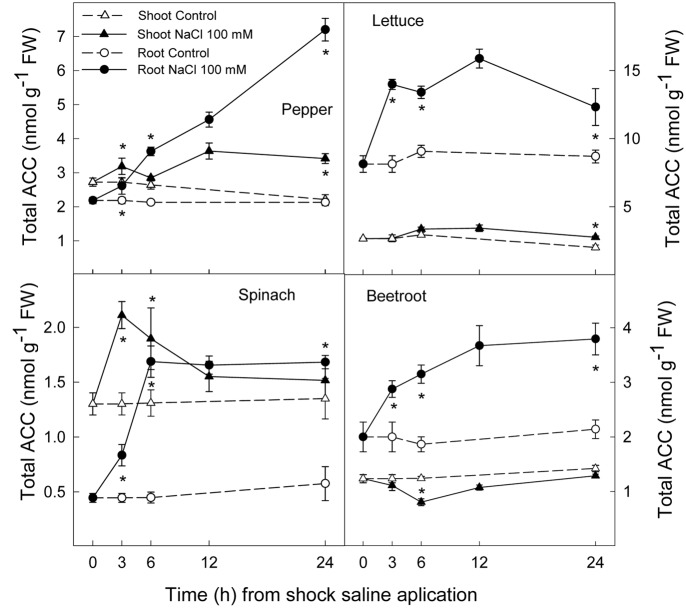
**Effect of saline shock (100 mM NaCl) on shoot and root total ACC concentration in different plant species**. Data are the mean ± SE of four replicates of three plants. ^∗^Shows significant differences between control and saline treatment for each sampling date.

Ethylene production increased rapidly in shoot and root in all plant species after the saline shock application. In shoot, this increase in ethylene production tended to decrease in the least salt sensitive species, in which similar levels to those of control plants were reached after 12–24 h. Moreover, in beetroot shoot the lowest increase in ethylene production due to salinity was found. Accordingly, in root ethylene production was significantly increased by saline shock in the four plant species, this effect being evident after 3 h. Ethylene continued increasing after 24 h in the two more salt sensitive species, while in the least sensitive ones a decrease occurred after 12 h. At the end of the experiment in beetroot shoot and root ethylene production was similar in both control and treated plants (**Figure 5**). The increase in ethylene production was due to a significant enhancement in ACC concentrations, that is both free ACC and total ACC, which were detected after 3 h of saline shock, except in shoot of beetroot, in which free ACC and even total ACC decreased after saline shock treatment (**Figures 6**, **7**). Ethylene production after 24 h remained higher in salinity-treated than control plants in shoot and root of pepper and lettuce, while in spinach this occurred only in root. No differences were observed in beetroot. Thus ethylene production was related to the higher free ACC concentration found in shoot and root of saline stressed plants, except in the root of beetroot, in which ethylene production was similar in control and stressed plants after 24 h in spite of the higher free ACC in the latter.

### Polyamine Levels

**FIGURE 8:**
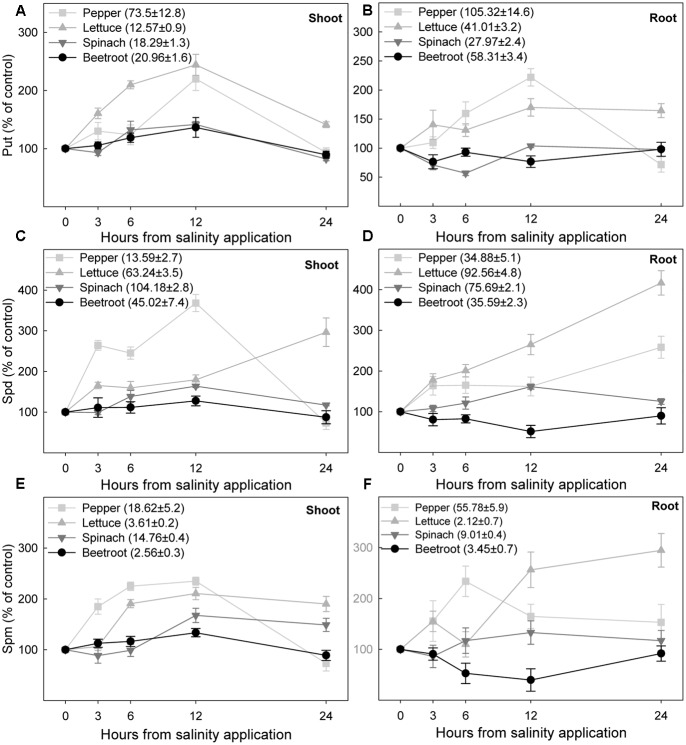
**Changes in putrescine, spermidine, and spermine under saline stress (expressed as percentage with respect to controls) in shoot (A,C,E)** and root **(B,D,F)** of the different plant species, after saline shock application. Data are the mean ± SE of determinations made independently in four replicates of three plants. Data in brackets are the value of each polyamine before the application of saline shock.

The initial endogenous levels of Put before salinity application differed amongst the species, being the following in shoot and root for each species in nmol g^-1^ FW: 73.1 ± 7.8 and 105.3 ± 14.7 in pepper, 12.5 ± 0.9 and 41 ± 3.2 in lettuce, 18.3 ± 1.3 and 28 ± 2.4 in spinach, and 21 ± 1.7 and 58.3 ± 3.4 in beetroot. In the two most sensitive species the application of 100 mM NaCl induced a Put accumulation in shoot and root after 3 h which continued until 12 h after the treatment, when the highest concentration was reached, 120–140% increase in shoot and 70 and 122% increase in root from lettuce and pepper, respectively (**Figures 8A,B**). Put concentration then decreased, being similar to control values after 24 h, except in roots from lettuce seedlings. In the two most salinity-tolerant species, spinach and beetroot, salinity only slightly increased Put level in shoot 6 and 12 h after the NaCl application. In root Put levels slightly decreased after 3–6 h of salinity application.

Endogenous levels of Spd in shoots and roots under control conditions differed in the four studied species, with the lower levels being found in pepper and beetroot (from 10 to 45 nmol g^-1^ FW), while in lettuce and spinach the values ranged from 76 to 104 nmol g^-1^ FW. In most cases Spd changes due to salinity were higher than Put changes. Thus, Spd increased 3 h after stress in shoot and root of pepper and lettuce seedlings, reaching a maximum increase of 317% in lettuce root at 24 h, followed by pepper shoot in which Spd increased 168% at 12 h although similar levels to controls were reached after 24 h (**Figures 8C,D**). In spinach shoot and root Spd increased after 6 h of stress, but to a lesser extent than in pepper and lettuce. Contrarily, in the root from beetroot salinity had the opposite effect, decreasing Spd concentration. In the most tolerant species, spinach and beetroot, 24 h after stress imposition levels of Spd were similar to those under control conditions.

Spermine was found at a lower concentration than Put and Spd, ca. 20 and 50 nmol g^-1^ FW in shoot and root of pepper respectively, between 9 and 15 nmol g^-1^ FW in spinach and ranged from 2 to 4 nmol g^-1^ FW in lettuce and beetroot. In shoots from pepper and lettuce Spm increased from 3 h after stress, and in spinach and beetroot from 6 to 12 h. The maximum Spm level was reached at 12 h, being an increase of 135% for pepper, 111% for lettuce, 68% for spinach, and 34% for beetroot. Salinity also increased the level of Spm in roots. However, this effect was lower than for shoots (**Figure 8E**), and even in beetroot Spm was decreased by salinity. Accumulation of Spm tended to stabilize with time, except in lettuce roots (**Figure 8F**).

**FIGURE 9:**
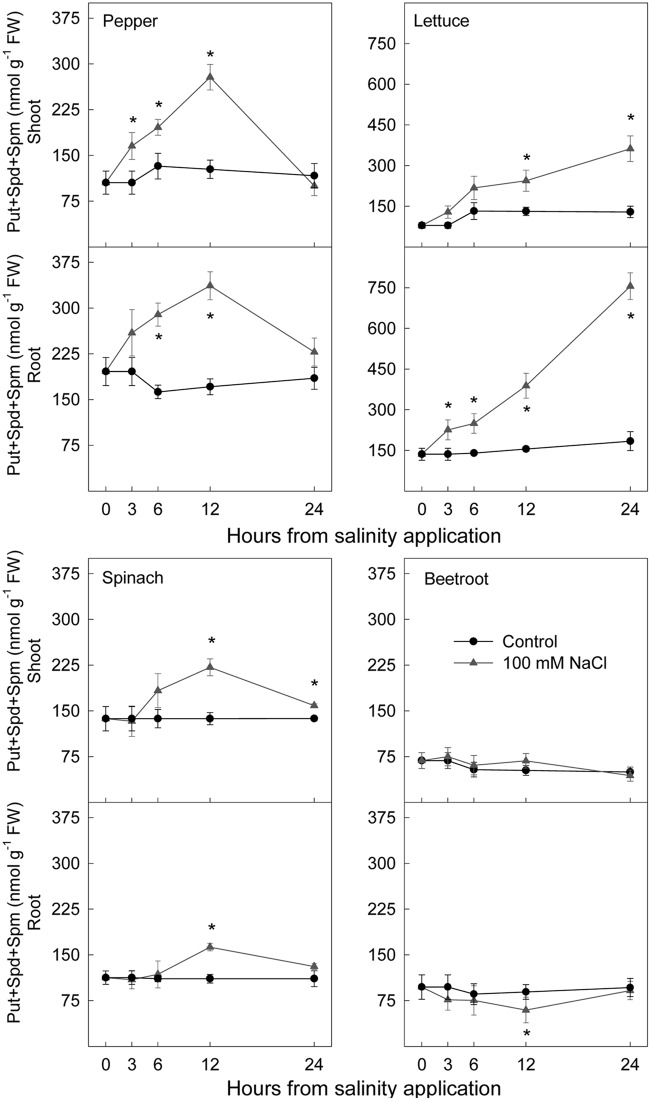
**Effect of saline shock on total polyamine concentration, in shoot and root of the different plant species**. Data are the mean ± SE of determinations made independently in four replicates of three plants. ^∗^Shows significant differences between control and saline treatment for each sampling date.

Total polyamine concentration (**Figure 9**) increased markedly after saline stress imposition in shoots and roots of the two most sensitive species, pepper and lettuce. This increase started 3 h after stress imposition. In spinach total polyamine content increased to a lesser extent than for these two species in shoot and root, the increase occurring after 6–12 h. In beetroot, no effect of salinity in shoot and a decrease of total polyamine in root were found.

## Discussion

The effect of saline shock on reducing fresh weight is attributed to the osmotic effect of salinity reducing water uptake (Munns, 2002). Our results showed that the osmotic component of salinity provoked an osmotic shock which was related to saline sensitivity, in agreement with Lefèvre et al. (2001). In this sense, the sudden decrease in water potential of nutrient solution when saline treatment was applied led to a decrease in water content in shoot and root of the four plant species, which was observed after 3 h of treatment. However, water content in spinach and beetroot was completely recovered after 24 h of stress while lettuce and pepper shoots and roots did not totally recover the water content of control plants after 24 h. Thus the latter species behaved as more sensitive to saline shock than spinach and beetroot according to previous experiments with long term saline treatments (Zapata et al., 2007, 2008).

In general, saline shock caused a sudden increase in respiration rate in all plant species, although it tended to disappear in the most tolerant, beetroot, while remaining after 24 h in the most salt sensitive. Accordingly, during germination and early seedling development, salinity has been shown to lead to increased respiration rate in a wide range of plant species, its magnitude being related to plant saline sensitivity (Zapata et al., 2004).

Ethylene is a plant hormone having a role in plant responses to biotic and abiotic stresses including salt stress (Ryu and Cho, 2015; Tao et al., 2015). Saline stress led to a quick increase in ethylene production in all plant species, although it tended to recover to initial levels in the most tolerant and remained at higher levels after 24 h in the most sensitive species. Accordingly, free and total ACC increased as a consequence of saline shock after 3 h of treatment. Levels still remained high at the end of the experiment in all plant species, except in beetroot shoot, in which free and total ACC levels were lower in treated than in control plants. Thus, in pepper, lettuce and spinach, a sharp increase in ACS activity occurred as a consequence of saline shock, while in the most tolerant species, beetroot, ACS activity in shoot seemed to be reduced as a consequence of saline shock, since the initial total-ACC levels decreased after treatment. Similar results were found when salinity was applied in a progressive way to these plant species and analysis performed after several days. Here a continuous increase in ACC concentration was found in the most sensitive plants, while in spinach this increase was lower and in beetroot no increase occurred (Zapata et al., 2007). In addition, it is also important to note that in shoot and root of pepper and lettuce plants and in spinach and beetroot shoot the salt induced increase in total ACC was due to both free ACC and conjugated ACC, since in tissues total ACC was higher than free ACC. However, in root of spinach and beetroot, the activity of malonyl-ACC transferase (the main enzyme responsible for ACC conjugation) was very low or zero in control and saline conditions. This is recognized because similar levels of total and free ACC were found. Moreover, in root of beetroot free ACC was higher with salinity after 24 h while ethylene production was similar in control and treated plants, showing an inhibition of ACC oxidase activity in the stressed plants.

Accordingly, in wheat, corn, and soybeans cultivars it has been reported that saline treatment led to higher ethylene production in sensitive cultivars than in more tolerant ones (Lu et al., 1991; Pennazio and Roggero, 1991; El-Shintinawy, 2000). However, in lettuce cultivars saline treatment applied during germination phase increased seedling ethylene production, although its magnitude was higher in the most tolerant cultivars, suggesting that ability to increase ethylene production under saline conditions could provide a higher tolerance to salinity during germination (Zapata et al., 2003). It is not clear if ethylene is involved in the acclimatization processes that aid plants to survive or if it is just a stress response. In this sense, the role of ethylene signaling in plant tolerance to salinity should be considered. Recently, it has been claimed that the ability of plants to retain K^+^ and/or a large K^+^/Na^+^ ratio is more important than the efficient exclusion of Na^+^ from shoots and roots or the compartmentalisation in special tissues or cells (Møller et al., 2009; Shabala et al., 2010). In fact, ethylene signaling has been found to be essential for both plant tolerance to salinity (Cao et al., 2007; Wang et al., 2007, 2009) and maintenance of high K^+^ content (Jung et al., 2009). Moreover, in experiments performed with Arabidopsis it has been found that ethylene insensitive mutants were more affected by salinity than wild type plants, salt tolerance being correlated with plant ability to retain K^+^ in roots and/or shoots (Yang et al., 2013).

Using whole plant systems of four plant species our results clearly show that the short-term effect of saline shock caused immediate increases in the levels of Put, Spd, and Spm in all cases in shoots, and in some cases in roots, and that those changes were related to the salinity sensitivity of the plants. In fact, in the two most sensitive species, pepper and lettuce, saline stress induced in shoot and in root a higher Put, Spd, and Spm increase than in the species considered as more tolerant, i.e., spinach and beetroot, with the latter demonstrating the smallest increase in total polyamine content. These polyamine increases were related to the highest osmotic effect (measured by water content decrease) that occurred in shoot and root from pepper and lettuce seedlings. The physiological response induced by salinity in polyamine synthesis was rapid (3 h in the most sensitive species, and 6 h in the most tolerant ones), thus polyamine may be implicated in the adaptation response to stress. However, in most cases changes in polyamines were transitory and polyamine level decreased 24 h after stress to similar levels to those of the control (except for lettuce seedlings). Similar results have been found by other authors, such as Aziz and Larher (1995) and Mutlu and Bozcuk (2005), suggesting that when salinity is applied the polyamine biosynthesis increases, but levels rapidly begin to decrease in parallel to the accumulation of ions. However, in the study from Tonon et al. (2004) with embriogenic *Fraxinus angustifolia* callus, similar results occurred in response to mannitol. Therefore, the decrease on polyamines may not be associated only with Na^+^ accumulation. Similarly, Legocka and Kluk (2005) found in lupinus that prolonged (24 h) salt and osmotic stress conditions resulted in a decline in Put and Spd in root, the authors indicating that the polyamine biosynthetic pathway may be sensitive to high salinity and/or water deficit with longer exposition to both stresses, causing the change from biosynthetic processes to oxidative degradation.

Considering the idea of a reduction of polyamine levels with time related to accumulation of saline ions Na^+^ and Cl^-^ with time, results were different in the four considered species. In the most tolerant species, spinach and beetroot, there was an accumulation of Na^+^ in shoot that was higher than in the two sensitive ones (pepper and lettuce) and as such the increase in polyamines due to salinity was lower than in the sensitive ones. Thus, those species accumulating more Na^+^ and Cl^-^ in shoot, and more salinity tolerant, can use those ions for osmoregulation and changes in polyamines as a consequence of saline stress are lower than in the sensitive ones. These results confirm previous experimental findings (Zapata et al., 2008) about the long term effect of salinity on the same species. However, in the species considered more sensitive to salinity, pepper and lettuce, results were different. They accumulated high Na^+^ concentration in root and showed a limitation for Na^+^ accumulation in shoots while presenting the highest polyamine increase. The decrease in polyamines in pepper shoot after 24 h of exposition to stress cannot be explained by an accumulation of the saline ions Na^+^ or Cl^-^ (they did not accumulate in shoot). It may be considered that the low accumulation of Na^+^ and Cl^-^ was enough to decrease polyamine levels. In the case of lettuce, Na^+^ and Cl^-^ accumulation was high in root, but at 24 h there was not observed a decrease in polyamine levels in that plant part. It could be argued that in lettuce a period longer than 24 h is needed in order to observe a decrease in polyamines.

When the long-term effect of salinity (days) was studied in the same plant species (Zapata et al., 2008) the changes in polyamine levels were low and even decreased, probably due to plant adaptation to that stress. Therefore, our results would agree with those from Das et al. (1995), who found in *Brassica campestris* that polyamine levels and related enzymatic activities were little affected by long-term stress, but significantly increased by short-term stress. Moreover, in tomato polyamine levels were affected by the length or duration of the stress (Botella et al., 2000).

In the literature there are some studies focused on comparing closely related salt sensitive and salt tolerant species or cultivars in relation to the accumulation of polyamines. Interestingly, in rice and tomato the accumulation of polyamines was greater in salt sensitive than in salt tolerant lines (Katiyar and Dubey, 1990; Aziz et al., 1999). Our results in different plant species with different tolerance to salinity clearly indicate that the increase in polyamines was higher in the two most sensitive species, pepper and lettuce, showing that a higher increase in polyamines would indicate a higher level of stress. Similarly, the study from Minocha et al. (2014) revealed that foliar Put concentration could be used as a reliable biochemical marker for early detection of stress due to soil Ca^2+^-deficiency in natural forests before the appearance of any visual symptoms of stress damage. These results would support the idea from Jouve et al. (2004) who suggested that in order to select plants for their ability to sustain satisfactory yields under saline conditions, the characterization of several compounds of the metabolome of an organism during exposure to a stress can be a complementary approach to studies of changes in the transcriptome and proteome, which are nowadays generally used as markers.

In spite of the fact that pathways of ethylene and polyamines (Spd and Spm) biosynthesis share SAM as a common precursor, they exert opposite effects in plant development processes. Thus, reduced levels of polyamines have been correlated with increased ethylene production and senescence, while high endogenous concentrations of polyamines are associated with a delay in this process. In this sense, the balance between these two opposite growth regulators is crucial to retard or to accelerate senescence and plant responses to environmental factors and even competence for SAM has been claimed to occur (Sauter et al., 2013; Tiburcio et al., 2014; Serrano et al., 2016). However, in the four plant species studied increases in ethylene, ACC, Spd, and Spm were found as a consequence of the saline treatment, showing that no competence between them occurred and that the SAM pool is high enough to support both ethylene and polyamines biosynthesis.

We can conclude that under saline shock plants respond with increases in ethylene production and polyamine concentration, these being related to plant sensitivity to this stress. The magnitude of these increases was higher and occurred earlier in pepper and lettuce, the most salt sensitive species. Ethylene production decreased after 24 h in salt tolerant plants while still remaining high in the most sensitive. Increases in polyamines tended to disappear after 24 h, except in lettuce. Thus, ethylene and polyamines may have a role as a stress signal once increasing they might induce adaptive responses to the stress. In addition, no competition for the common precursor, SAM, was observed between ethylene and polyamines biosynthetic pathways.

## Author Contributions

MB has participated in the design of the experiments, the analytical determinations and the writing and discussion of the manuscript. MS has participated in the design of the experiments, the analytical determinations and the writing and discussion of the manuscript. MP has participated in the design of the experiments, the analytical determinations and the writing and discussion of the manuscript. PZ has participated in the design of the experiments and the analytical determinations. MG-L has participated in the design of the experiments and the analytical determinations. All authors revised the manuscript.

## Conflict of Interest Statement

The authors declare that the research was conducted in the absence of any commercial or financial relationships that could be construed as a potential conflict of interest.
